# Impact of the affordable care act on utilization of benefits of eye care and primary care examinations

**DOI:** 10.1371/journal.pone.0241475

**Published:** 2020-11-02

**Authors:** Yi Pang, Zhiyong Ren, Jingyun Wang

**Affiliations:** 1 Illinois College of Optometry, Chicago, IL, United States of America; 2 SUNY College of Optometry, New York, NY, United States of America; University of Utah, UNITED STATES

## Abstract

**Purpose:**

To determine the impact of the Affordable Care Act (ACA) on utilization of benefits of both eye care and primary care examinations in individuals who did not have health insurance prior to the ACA.

**Methods:**

Patients examined in an urban eye clinic from 2017 to 2018 were invited to participate. Patients were classified into two groups: Insured Group, who had health insurance before and after the ACA; The ACA Group, who had insurance only after the ACA. Patients were surveyed on how often they were examined by their eye care and primary care physicians before and after the ACA. The care utilization frequency was categorized into 3 levels: Frequent Care Use, Rare Care Use, and Never. To test the utilization of benefits frequency difference between two groups, the z-ratio was calculated.

**Results:**

A total of 4,355 patients were enrolled with 87.1% in the Insured Group and 12.9% in the ACA Group. After the ACA implementation, the percentage of “Frequent Care Use” of the eye care and primary care in the ACA Group patients significantly increased from 31.2% and 53.7% to 57.9% and 74.9%, respectively (P<0.001), but were significantly lower than those in the Insured Group (76.6% and 93.9%, P < 0.001).

**Conclusion:**

The ACA significantly improved utilization of benefits of eye care and primary care for individuals in the ACA Group. Although improved, those patients who received health insurance through the ACA still had lower utilization of benefits than those in the Insured Group.

## Introduction

The Patient Protection and Affordable Care Act (ACA) was signed into law in March 2010. Since then, approximately 20 million US residents have gained health insurance [1]. Since the implementation of ACA, many claims have been stated on both sides regarding the ACA’s success or failure [2–7].

The ACA has three primary goals: increasing the number of insured, improving the quality of care, and reducing the costs of health care. Although the first goal has been clearly reached [2, 3], several studies have reported that the ACA has not improved the quality of care [4, 5, 7]; in addition, it was suggested that access to health care seems to have diminished [4, 5, 7]. Health insurance is not health coverage. Health insurance is a financial mechanism of paying for health care, whereas coverage refers to the health care services provided under that insurance and received by patients [8]. Whether patients who historically had no health insurance coverage prior to the ACA actually use the benefit is a key factor in health care quality.

Use of eye care is critical to individual’s health-related quality of life and is associated with other health care burdens and costs. Among people aged over 40 years, moderate/severe visual impairment was strongly associated with poor health-related quality of life [9, 10]. Compared with those without vision loss, patients with severe vision loss who are hospitalized for common illnesses are often associated with 4% longer lengths of stay, 22% higher odds of readmission, and 12% higher costs [11]. One would expect that health insurance coverage should help improve patients’ visiting frequency of both eye care and primary care examinations. However, to the best of our knowledge, the impact of the ACA on patient utilization of benefits in eye care has not been reported.

The aim of this prospective study was to determine the impact of the ACA on patient utilization of benefits of both eye care and primary care examinations in individuals who historically had no health insurance prior to the ACA. We hypothesized that ACA improved patient utilization of both eye care and primary care to the same level as individuals who had health care before the ACA.

## Methods

### Institutional review board approval

The research protocol (protocol number: 16033) and informed consent forms were approved by the Institutional Review Board (IRB) of Illinois College of Optometry (Chicago, IL). Adherence to the Health Insurance Portability and Accountability Act (HIPAA) was maintained during this study. In accordance with the guidelines of the Declaration of Helsinki, appropriate informed consent was obtained from the participants and/or the parents or guardians of participants younger than 18 years old.

### Participants

This study was conducted at an urban eye clinic of the Illinois Eye Institute (Chicago, IL) which provides both primary and secondary/tertiary eye care by both optometrists and ophthalmologists. All patients examined from April 1, 2017 to January 31, 2018 (N = 31,124) were invited to participate in this study. Participants ranged from birth to 64 years of age. A total of 4,355 participants were enrolled into the study with a participation rate of 14.0%.

According to the patients’ self-reported insurance status, patients were classified into two groups: 1) Insured Group: patients who had health insurance before and after the ACA. 2) The ACA Group: patients who did not have health insurance prior to the implementation of ACA and ONLY received health insurance under the ACA implementation.

### Description of procedures and outcome variables

The questionnaire was developed to determine how often individuals had their eye care and primary care examinations before and after the implementation of ACA (**S1 Data**) [12, 13]. Regarding the frequency of doctor visits, the following options were provided to the participants: *more than once a year*, *once in 1 to 2 years*, *once in 3 to 5 years*, *once in 5 to 10 years*, *never*, *not sure*. The benefits utilization frequencies were categorized into 3 levels:

1. *Frequent Care Use*, if the answer key is “more than once a year” or “once in 1 to 2 years”;
2. *Rare Care Use*, if the answer key is “3 to 5 years”, or “5 to 10 years” or “not sure”;
3. *Never*, if the answer key is “never”.

### Statistical analysis

Data analysis was conducted using SPSS software [Version 24, IBM®SPSS, Inc, N.Y., United States (US)]. Descriptive statistics were applied to data and the calculated proportion of benefits utilization. T-tests were used for analysis of continuous variables. To test the proportion of benefits utilization difference between the Insured Group and the ACA Group, the z-ratio was calculated for the significance of the difference between two independent proportions. A P-value of < 0.05 was considered statistically significant. Because the change in “frequent care use” indicated impacts of the ACA on utilization of health insurance, the relationship of gender, race, and age subgroups with “frequent care use” was determined by Chi-square test. Age was classified into 3 subgroups: children (<18 years old), young adults (between 18 and 45 years old), and middle-aged adults (between 45 and <65 years old).

## Results

In the total of 4,355 enrolled participants, 87.1% (N = 3,794) had health insurance before and after the ACA (Insured Group) and 12.9% (N = 561) received health insurance under the ACA implementation (ACA Group).

The sex, age, and race/ethnicity characteristics of the participants are listed in **Table 1**. The mean age of participants in the two groups was significantly different (t-test = 3.5. P<0.001). The average age of the Insured Group was about one year younger than the ACA Group. In this sample, 60% of our participants were African American and 27% of our participants declined to report their races/ethnicity.

**Table 1:** Demographic characteristics of the participants (N = 4,355).

	Total N = 4355	ACA Group N = 561	Insured Group N = 3794	P-Value
Sex				
Female No. (%)	2816 (65)	275 (49)	2541 (67)	<0.001* ^1^
Male No. (%)	1539 (35)	286 (51)	1253 (33)	
Race/Ethnicity				
African American No. (%)	2632 (60)	306 (55)	2326 (61)	0.08 ^2^
Asian No. (%)	101 (2)	18 (3)	83 (2)	
Caucasian No. (%)	156 (4)	20 (4)	136 (4)	
Hispanic No. (%)	282 (6)	61 (11)	221 (6)	
Other No. (%)	23 (1)	1 (0)	22 (1)	
Declined No. (%)	1161 (27)	155 (28)	1006 (27)	
Age (year)				<0.001*
Mean (SD)	41.69 (17.47)	43.90 (15.84)	41.36 (17.67)	
Age≤18 No. (%)	617 (14.1)	45 (8)	572 (15.1)	<0.001*
Age 19 to <45 No. (%)	1347 (30,9)	187 (33.3)	1160 (30.6)	0.20
Age 45 to <65 No. (%)	2391 (54.9)	329 (58.6)	2062 (54.3)	0.06

* Indicates statistical significance between the ACA Group and the Insured Group.

^1^ The significance of the difference between the proportions of female in the two groups was tested by the Chi-squared (Chi-squared = 68.1).

^2^ The significance of the difference between the proportions of African American in the two groups was tested by Chi-squared (Chi-squared = 1.7).

^3^ The significance of the difference between the proportions of age subgroups in the two groups was tested by Chi-squared (Chi-squared = 19.4, 1.6, 3.5 for three age subgroups).

**Fig 1:**
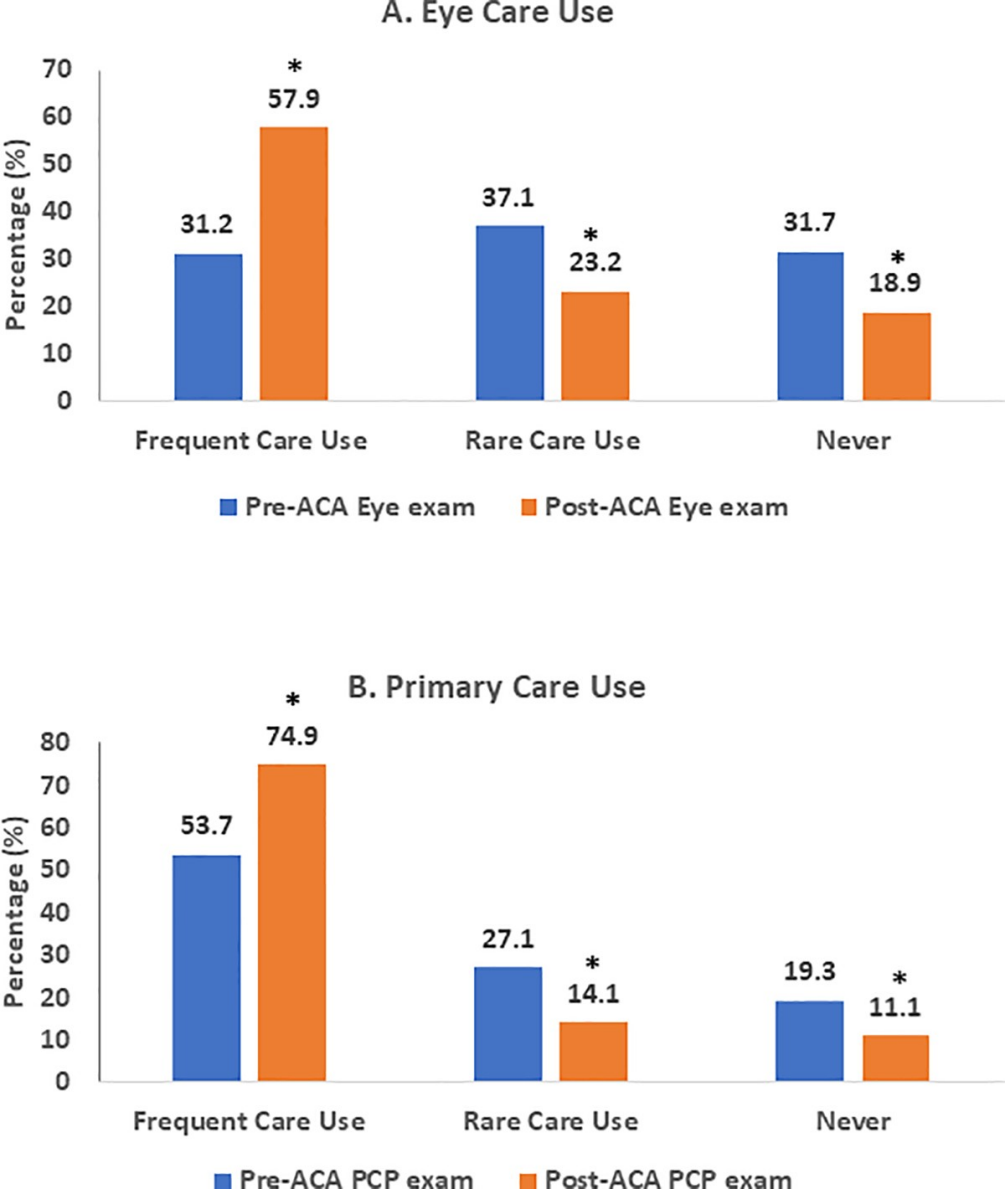
**A.** Utilization change of eye care in the ACA Group. **B.** Utilization change of primary care in the ACA Group. Asterisk indicates statistical significance in utilization of eye care and primary care compared post-ACA with pre-ACA.

**Fig 1A & 1B** demonstrates the percentage of utilization of benefits of Eye Care and primary care in the two groups. Prior to the ACA, only 31.2% and 53.7% of participants in the ACA Group had “*Frequent Care Use”* from eye care and primary care, respectively, vs. 76.6% and 93.9% in the Insured Group participants with statistically significant differences (both P-values < 0.01). After the implementation of ACA, the percentage of “*Frequent Care Use*” with Eye Care in the ACA Group improved significantly (from 31.2% to 57.9%, P<0.01). The percentage of “*Frequent Care Use*” with primary care in the ACA Group also improved significantly (from 53.7% to74.9%, P<0.01).

Although the percentage of “*Frequent Care Use*” in the ACA Group significantly improved after the ACA implementation, it was still significantly lower than those of the Insured Group (76.6% in Eye Care and 93.9% in primary care, both P-values <0.01). Furthermore, the relationship between the category of “Frequent Care Use” with gender, race and age subgroups was determined (**Table 2**). For the insured group, utilization of both eye care and PCP was statistically significantly higher in females than in males (both P-values < 0.05); African American had statistically significantly higher utilization of primary care than other races (P <0.001); children had higher utilization of both eye care and primary care than young adults and middle-aged adults (both P values < 0.001). For the ACA group, a statistical significant increase in utilization of eye care was found in males after the ACA (P < 0.05); a statistical significant increase in utilization of both eye care and primary care was found in young adults (both P-values < 0.01).

**Table 2:** Percentage of utilization of benefits of eye care and primary care exams (%) in the two groups stratified by gender, race and age subgroups among respondents reporting “frequent care use”.

Percentage(%)	Insured Group (N = 3794)				ACA Group (N = 561)					
	Eye Exam	^1^P-Value(Chi-squared)	Primary Care Exam	^1^P-Value (Chi-squared)	Eye Exam			Primary Care Exam		
					Before ACA	After ACA	^2^P-Value (Chi-squared)	Before ACA	After ACA	^2^P-Value (Chi-squared)
Female	77.6	<0.05*	95.0	<0.05*	37.4	60	<0.05*	57.8	78.5	0.86
Male	74.4	(4.5)	91.6	(15.7)	25.1	55.9	(4.4)	49.6.1	71.3	(0.03)
African American	76.5	0.97 (0.001)	95.0	<0.001 (12.4)	33	58.5	0.52 (0.4)	56.2	78.1	0.74
										(0.11)
Other races	76.6		92.1		29	57.2		50.6	71.0	
Children	81.4	<0.001*	96.3	<0.001*	26.7	46.7	<0.01*	40	48.9	<0.01*
Young adults	69.8	(43.8)	90.6	(32.1)	32.2	64.4	(12.2)	56.5	82	(10.2)
Middle-aged	79.0		95.0		30.5	49.2		51.9	68.4	

^1^ The significance of the difference between the proportions of gender, race, and age subgroups with “frequent care use” was tested by the Chi-squared.

^2^ The significance of the difference between the difference of before and after proportions of gender, race, and age subgroups with “frequent care use” was tested by the Chi-squared.

Accordingly, after the ACA implementation, the percentage of “*Rare Care Use*” and “Never” in the ACA Group significantly decreased for both Eye Care and PCP Care, however it was still significantly higher than those in the Insured Group (**Table 3**).

**Table 3:** Percentage of utilization of benefits of eye care and primary care exams (%) in the two groups.

Percentage (%)	Insured Group (N = 3794)				ACA Group(N = 561)					
	Eye Exam	P-Value	Primary Care Exam	P-Value	Eye Exam			Primary Care Exam		
					Before ACA	After ACA	P-Value	Before ACA	After ACA	P-Value
Frequent Care Use	76.6 *	<0.01	93.9 *	<0.01	31.2	57.9 #	<0.01	53.7	74.9 #	<0.01
Rare Care Use	19.5 *	<0.05	5.5 *	<0.01	37.1	23.2 #	<0.01	27.1	14.1 #	<0.01
Never	4.0 *	<0.01	0.7 *	<0.01	31.7	18.9 #	<0.01	19.3	11.0 #	<0.01

# The percentage in the ACA Group after the ACA implementation was significantly different compared with that before the ACA implementation.

* After the ACA implementation, the percentage in the ACA Group was significantly different from the Insured Group.

## Discussion

To our knowledge, our study is the first one to report patient utilization of Eye Care after the ACA implementation. The ACA generally mandates vision insurance for children under age 19. Although vision insurance is not covered by the ACA for adults, medically necessary eye exams are covered under the ACA.

In this study, over half of our participants were African Americans, and over a quarter of our participants declined to report their races, which hinders race/ethnicity disparity analysis. The ACA is aimed at eliminating racial/ethnic and other health disparities by improving health care access and quality and by more accurately tracking health disparities [14].

Prior to the ACA, without insurance, only about 31% of the ACA Group reported *Frequent Care Use* in eye care and 58% of participants received primary care. In contrast, the Insured participants reported *Frequent Care Use*—77% in eye care and 94% in primary care. After the ACA implementation, the percentage of “*Frequent Care Use*” in the ACA Group significantly improved. Similar results on improved use of benefits after the ACA implementation have been reported in mental health care [15] and emergency visits [16, 17].

Females had higher utilization of both eye care and primary care in both groups in this study, which is consistent with previous studies [18, 19]. Interestingly, the ACA significantly increased the utilization of eye care and primary care in males although this was still lower than that in females. Another interesting finding in this study was that the ACA significantly increased utilizations of both eye care and primary care in young adults. Saloner and Le Cook reported that the ACA increased treatment for young adults with possible mental illnesses [20], which may be due to the ACA expanding health care coverage of young adults under parents’ health insurance. The same reason could help explain the findings in our study.

The percentage of “*Frequent Care Use*” in the ACA Group was still significantly lower than that of the Insured Group. This may indicate that the ACA participants are still not fully using their health insurance benefits, or other strategies need to be searched and implemented to improve patient care use besides health insurance. Our results confirm the previous report that the ACA implementation is slow and uneven, with incomplete take-up rates of eligible individuals [21]. Specifically, we expect a gradual move toward universal participation in the ACA though enhancing patients’ action on using benefits.

### Limitations

1) Data in this study were from self-reported answers, therefore, like other self-reported studies, this study might not avoid human errors from memory or misunderstanding. Information from an insurance database may reflect reality more precisely. 2). Selection bias may exist since this is a one-site study. Our findings may not generalize to other areas of the US. 3) We studied patients who visited the Illinois Eye Institute and individuals who did not utilize any care were missing from this study. 4) Our study only reflected the ACA implementation period before Feb. 2018. It is still actively ongoing and more impact may be observed after the study period. 5) Only 12.9% of our participants received health insurance under the ACA. Approximately 1 million (7.9%) out of all Illinoisans (12.67 million) have gained health care insurance under the ACA [22]. Although our study included a higher percentage of the ACA care beneficiaries than the general population in Illinois, the majority of our responders did not gain health insurance under the ACA. 5) It has been reported that external survey rate averaged about 10–15% [23]. There were 14% of the patients who asked to answer survey finished all survey questions, which matches the reported survey rate. However, our response rate could be increased by reminding participants multiple times. Participants were only approached once, and no reminder was sent in this study.

Even after almost a decade of implementation of the ACA, compared with our respondents who have health insurance, those in the ACA Group still have significantly lower utilization of eye care and primary care. It indicates that this population is not fully using equal health care. This phenomenon is also observed in other studies [24].

## Conclusion

The recent passage of the ACA has resulted in significant changes to the number of US residents with insurance coverage. The ACA expanded patient insurance coverage and significantly improved utilization of eye care and primary care. Even after almost a decade of implementation of the ACA, compared with our respondents who have health insurance, those in the ACA Group still have significantly lower utilization of eye care and primary care. It indicates that this population is not fully using equal health care. There is a long way to go.

## Supporting information

S1 Appendix Survey of frequencies of utilization of health insurance benefits of eye care and primary care exams (Survey version for patients aged 18 or older). (DOCX)

S1 Data (XLSX)
