# Supplementary material for: Impact of the affordable care act on utilization of benefits of eye care and primary care examinations
Source: PLoS One. 2020 Nov 2;15(11):e0241475. doi: 10.1371/journal.pone.0241475 (PMC7605705; doi:10.1371/journal.pone.0241475)
Supplement: S1 Appendix — (DOCX) [file pone.0241475.s001.docx]

**S1 Appensix: Survey of frequencies of utilization of health insurance benefits of eye care and primary care exams (Survey version for patients aged 18 or older)**

**
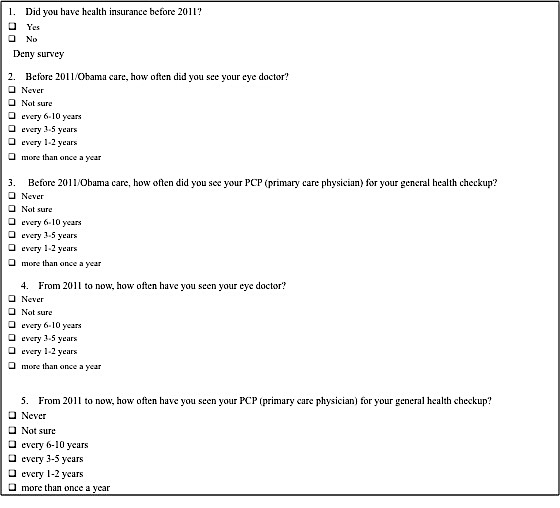
**
